# Integrative analysis of genetic, biochemical, and microbial factors in camel calf diarrhea

**DOI:** 10.1007/s11259-025-11027-w

**Published:** 2026-02-06

**Authors:** Ahmed Elsayed, Amani A. Hafez, Mohamed T. Ragab, Marwa A. Fawzy, Adel M. El-Kattan, Ahmed I. Ateya

**Affiliations:** 1 Department of Animal Health and Poultry, Animal and Poultry Production Division, Desert Research Center (DRC), Cairo, 11753 Egypt; 2 Department of Development of Animal Wealth, Faculty of Veterinary Medicine, Mansoura University, Mansoura, 35516 Egypt

**Keywords:** Camels, Biochemical profile, Diarrhea, Enteropathogens, Gene polymorphisms, Virulence gene

## Abstract

Diarrhea, though a clinical symptom, poses a major veterinary concern in camel calves due to high morbidity, mortality, growth retardation, and economic losses. This study investigated key bacterial enteropathogens, coding mutations, gene expression, and serum biochemical/antioxidant profiles in 35 healthy and 35 diarrheic camel calves. Fecal analysis using multiplex PCR identified *Salmonella spp.*, Clostridium perfringens, Proteus spp., and Escherichia coli with distinct virulence genes (e.g., papC, eaeA, iutA, hilA, sopB, plc, ureC). Diarrheic calves showed significant upregulation of immune-related genes (IL-8, IL-17, TLR4, TNFα, PIN1, IER3, UBD, TECPR1) and downregulation of antioxidant markers (PRDX2, SOD3, CAT, Nrf2), alongside sequence variations. Biochemically, diarrheic calves exhibited elevated serum AST, ALT, ALP, urea, creatinine, potassium, and MDA, with reduced glucose, total protein, calcium, phosphorus, magnesium, sodium, chloride, and SOD. These findings highlight the multifactorial etiology of diarrhea, linking bacterial virulence with metabolic and oxidative stress alterations, providing insights for targeted prevention and therapeutic strategies in camel herds.

**Supplementary Information:**

The online version contains supplementary material available at 10.1007/s11259-025-11027-w.

## Introduction

The dromedary camel (*Camelus dromedarius*) is a one-humped species valued for its contributions to meat, milk, leather, racing, and transport, supporting food security and economic development in arid and semi-arid regions (Saidi et al. 2018; Suliman et al. 2019; Swelum et al. 2020; Tibary and El Allali 2020). Although once thought resistant to livestock diseases, camels are now known to be susceptible to various pathogens. Diarrhea is a leading cause of illness and death in camel calves, causing significant economic losses (Elmahallawy et al. 2023; Hussein et al. 2024; Noaman et al. 2024). The condition is multifactorial, linked to management, environment, nutrition, and infections by bacteria, viruses, protozoa, and parasites (Muktar et al. 2015; Delgado-González et al. 2019). Calves under six months are most vulnerable, with mortality rates reaching nearly 40% in Sudan (Ali et al. 2005).

*Escherichia coli*,* Salmonella enterica*,* Clostridium perfringens*, and *Proteus spp*. are key bacterial enteropathogens in livestock diarrhea (Aiello et al. 2016; Juffo et al. 2017; Haq et al. 2018). Pathogenic *E. coli* (DEC), particularly Shiga toxin–producing strains, are common in Egyptian ruminants but poorly studied in camels (Alizade et al. 2019; Shahein et al. 2021). *Salmonella* serovars Typhimurium and Enteritidis cause major global losses (Herikstad et al. 2002), with virulence genes invA and hilA (SPI-1, SPI-2) essential for detection and colonization (Marcus et al. 2000; Murugkar et al. 2003; Akbarmehr 2010). *C. perfringens*, an anaerobic spore-former (Lu et al. 2022), causes enterotoxemia through toxin genes (plc, beta, epsilon, iota, and tpeL) (Uzal et al. 2010; Fayez et al. 2021). *Proteus spp.*, though less frequent, can cause neonatal diarrhea via virulence genes ureC and rsbA, often acting opportunistically in polymicrobial infections of camel calves (Alchalaby et al. 2025).

Diarrhea causes dehydration, electrolyte imbalance, metabolic acidosis, and organ dysfunction, potentially leading to septicemia, cardiac failure, or renal damage (Taylor et al. 2017). Early diagnosis reduces mortality (McGuirk 2008). Blood metabolic profiles (BMP) indicate health and disease severity, often showing marked changes during diarrhea (Antunović et al. 2009; Salem et al. 2018). Genetic studies using SNPs help identify disease resistance markers, highlighting the role of host genetics in infection outcomes (Adams and Schutta 2010; Pierce et al. 2020; Wang et al. 2022; Sallam et al. 2024).

However, information on biochemical changes, antioxidants, SNPs, and gene expression linked to diarrhea in camel calves is limited. This study aimed to investigate major bacterial enteropathogens and their virulence factors in relation to diarrhea, focusing on how infection influences immune and antioxidant gene expression, single nucleotide polymorphisms (SNPs), and serum biomarkers of oxidative stress and metabolism. By analyzing both biochemical profiles and genetic markers, the study sought to clarify how pathogen-induced oxidative imbalance and host genetic variations contribute to the onset and severity of diarrhea in camel calves.

## Materials and methods

### Design

Seventy female camel calves (< 3 months, 75–100 kg) were studied at Shalateen Research Station, Desert Research Center, Ras Hederba Valley, Egypt (22°00′720″ N, 36°48′955″ E), an arid region near Sudan and the Red Sea with ~ 58.5 mm annual rainfall, temperatures averaging 22–35 °C, and relative humidity of 37–43% (Egyptian General Meteorological Authority). Livestock grazing is the main livelihood (Askar et al. 2014; Nassar 2020). Clinical evaluation followed (Constable et al. 2016). Calves were classified into two groups: control (CG, *n* = 35) with normal health, and diarrheic (DG, *n* = 35) showing diarrhea, emaciation, dullness, fever, and elevated pulse and respiration.

### Sampling collection

#### Fecal samples

Rectal swabs were collected from each calf using sterile Dacron swabs. Calves were restrained in sternal recumbency, the perineal area cleaned, and swabs inserted 5–10 cm into the rectum, rotated, and placed in transport media (Cary–Blair/Amies for culture; RNAlater or sterile tubes for molecular work). Separate swabs were used for each purpose. Bacteriological samples were stored at 4 °C and processed within 24–48 h; molecular samples were stored at 4 °C short-term or − 80 °C long-term. All were labeled and transported under cold chain with standard biosafety precautions.

#### Blood samples

Ten milliliters of blood were collected from the jugular vein of each animal into both plain and EDTA tubes. Plain tubes were centrifuged at 3000 rpm for 20 min at 37 °C to obtain serum, which was stored at − 80 °C until biochemical analysis. EDTA tubes were used for RNA extraction.

### Isolation, identification, and characterization of pathogens

Bacteriological examination was performed to identify bacterial causes of diarrhea in neonatal camel calves. *Escherichia coli*,* Salmonella spp.*,* Clostridium perfringens*, and *Proteus spp.* were isolated and identified using standard microbiological and biochemical methods.

### Isolation and identification of *Escherichia coli*

Fecal swabs were enriched in nutrient broth and incubated at 37 °C for 18–24 h, then streaked onto MacConkey agar to isolate Gram-negative lactose fermenters. Pink colonies were subcultured on HiCrome™ UTI agar, where *E. coli* appeared with dark purple coloration. Suspected colonies were confirmed using standard biochemical tests including indole, methyl red, Voges-Proskauer, citrate, urease, and triple sugar iron (TSI) following Gershwin (1990) and Koneman et al. (1992).

### Isolation and identification of *Salmonella spp*

Fecal samples were enriched in tetrathionate and Selenite-F broths at 37 °C for 16–18 h to recover *salmonella* and suppress competing flora. Enriched cultures were streaked onto XLD and *Salmonella-Shigella* (SS) agars, where *salmonella* appeared as colonies with black centers after 24–48 h incubation at 37 °C. Presumptive isolates were identified biochemically using the API 20E system (BioMérieux, France) and confirmed serologically by slide agglutination with polyvalent O and H antisera following the Kauffmann–White scheme (Herrera-León et al. 2007).

### Isolation and identification of *Clostridium perfringens*

Fecal samples (1–2 g) were inoculated into cooked meat medium and incubated anaerobically at 40 °C for 24 h. Enriched cultures were streaked onto blood glucose agar (1% glucose, 5% sheep blood) and re-incubated anaerobically for 24 h. *Clostridium perfringens* was identified by double-zone hemolysis, gram-positive rod morphology, and non-motility, with confirmation by standard biochemical tests (Koneman et al. 1992).

### Isolation and identification of *Proteus species*

Rectal swabs were pre-enriched in peptone water at 37 °C for 24 h, and then streaked onto MacConkey agar. Swarming on blood agar and non-lactose-fermenting pale colonies on MacConkey agar were used to identify *Proteus species*. Confirmation was done using biochemical tests including urease positivity, H₂S production, indole, methyl red, and citrate utilization outlined by Quinn et al. (2011).

#### PCR-Based bacterial detection

Fecal samples (~ 1 g) were homogenized in 9 mL peptone water and enriched overnight at 37 °C. DNA was extracted from 1 mL using the QIAamp DNA Mini Kit (Qiagen, Germany) with minor modifications. PCR was performed in 25 µL reactions containing 12.5 µL EmeraldAmp Max Master Mix, primers (Takara, Japan), DNA template, and nuclease-free water, using an Applied Biosystems 2720 thermal cycler. Primer details and conditions are in Table 1.

**Table 1:** Primers sequences, target genes, amplicon sizes and cycling conditions

Target gene	Primers sequences	Amplified segment (bp)	Primary denaturation	Amplification (35 cycles)			Final extension	Reference
				Secondary denaturation	Annealing	Extension		
*E. coli phoA*	CGATTCTGGAAATGGCAAAAG	720	94˚C 5 min.	94˚C 30 sec.	55˚C 40 sec.	72˚C 45 sec.	72˚C 10 min.	Hu et al. 2011
	CGTGATCAGCGGTGACTATGAC							
*Salmonella invA*	GTGAAATTATCGCCACGTTCGGGCAA	284	94˚C 5 min.					Oliveira et al. 2003
	TCATCGCACCGTCAAAGGAACC							
*C. perfringens alpha toxin*	GTTGATAGCGCAGGACATGTTAAG	402	94˚C 5 min.					Yoo et al. 1997
	CATGTAGTCATCTGTTCCAGCATC							
*Proteus atpD*	GTATCATGAACGTTCTGGGTAC	595	94˚C 5 min.					Bi et al. 2013
	TGAAGTGATACGCTCTTGCAG							
*E. coli papC*	tgatatcacgcagtcagtagc	501	94˚C 5 min.	94˚C 30 sec.	58˚C 40 sec.	72˚C 45 sec.	72˚C 10 min.	Wen-jie et al. 2008
	ccggccatattcacataa							
*E. coli iutA*	GGCTGGACATGGGAACTGG	300	94˚C 5 min.	94˚C 30 sec.	63˚C 40 sec.	72˚C 45 sec.	72˚C 10 min.	Yaguchi et al. 2007
	CGTCGGGAACGGGTAGAATCG							
*E. coli eaeA*	ATGCTTAGTGCTGGTTTAGG	248	94˚C 5 min.	94˚C 30 sec.	51˚C 30 sec.	72˚C 30 sec.	72˚C 7 min.	Bisi-Johnson et al. 2011
	GCCTTCATCATTTCGCTTTC							
*E. coli vat*	AACGGTTGGTGGCAACAATCC	420	94˚C 5 min.	94˚C 30 sec.	58˚C 40 sec	72˚C 45 sec.	72˚C 10 min	Boisen et al. 2009
	AGCCCTGTAGAATGGCGAGTA							
*Salmonella avrA*	CCT GTA TTG TTG AGC GTC TGG	422	94˚C 5 min.	94˚C 30 sec.	58˚C 40 sec.	72˚C 45 sec.	72˚C 10 min.	Huehn et al. 2010
	AGA AGA GCT TCG TTG AAT GTC C							
*Salmonella sopB*	tca gaa gRc gtc taa cca ctc	517	94˚C 5 min.	94˚C 30 sec.	58˚C 40 sec.	72˚C 45 sec.	72˚C 10 min.	
	tac cgt cct cat gca cac tc							
*Salmonella bcfC*	acc aga gac att gcc ttc c	467	94˚C 5 min.	94˚C 30 sec.	53˚C 40 sec.	72˚C 45 sec.	72˚C 10 min.	
	ttc tgc tcg ccg cta ttc g							
*Salmonella hilA*	CATGGCTGGTCAGTTGGAG	150	94˚C 5 min.	94˚C 30 sec.	60˚C 30 sec.	72˚C 30 sec.	72˚C 7 min.	Yang et al. 2014
	CGTAATTCATCGCCTAAACG							
*C. perfringens* NetB	GCTGGTGCTGGAATAAATGC	560	94˚C 5 min.	94˚C 30 sec.	58˚C 40 sec.	72˚C 45 sec.	72˚C 10 min.	Datta et al. 2014
	TCGCCATTGAGTAGTTTCCC							
*C. perfringens* tpeL	ATATAGAGTCAAGCAGTGGAG	466	94˚C 5 min.	94˚C 30 sec.	55˚C 40 sec.	72˚C 45 sec.	72˚C 10 min.	Bailey et al. 2013
	GGAATACCACTTGATATACCTG							
*C. perfringens* plc (α-toxin)	ATA GAT ACT CCA TAT CAT CCT GCT	283	94˚C 5 min.	94˚C 30 sec.	55˚C 30 sec.	72˚C 30 sec.	72˚C 7 min.	Akhi et al. 2015
	AAG TTA CCT TTG CTG CAT AAT CCC							
*Proteus rsbA*	TTGAAGGACGCGATCAGACC	467	94˚C 5 min.	94˚C 30 sec.	58˚C 40 sec.	72˚C 45 sec.	72˚C 10 min	Pathirana et al. 2018
	ACTCTGCTGTCCTGTGGGTA							
*Proteus ureC*	GTTATTCGTGATGGTATGGG	317	94˚C 5 min	94˚C 30 sec.	56.2˚C 40 sec	72˚C 40 sec	72˚C 10 min.	
	ATAAAGGTGGTTACGCCAGA							

Bacterial virulence genes were detected by PCR using specific primers. In *E. coli*, papC, iutA, and eaeA were amplified (Wen-jie et al. 2008; Yaguchi et al. 2007; Bisi-Johnson et al. 2011). For *Salmonella spp*., hilA, avrA, bcfC, and sopB were targeted (Yang et al. 2014; Huehn et al. 2010). *Clostridium perfringens* genes included plc, tpeL, and netB (Bailey et al. 2013; Datta et al. 2014; Akhi et al. 2015), while *Proteus spp*. were screened for ureC, atpD, and rsbA (Pathirana et al. 2018).

### Quantification, expression, and genetic variation of immunological and antioxidant genetic markers

Total RNA was extracted from EDTA blood using Trizol (RNeasy Mini Kit, Qiagen) and assessed by NanoDrop. cDNA was synthesized with RevertAid Kit (Thermo Fisher), and real-time PCR with SYBR Green (SensiFAST, Bioline) measured immune (IL-8, IL-17, TLR4, TNFα, PIN1, IER3, UBD, TECPR1) and antioxidant (PRDX2, SOD3, CAT, Nrf2) gene expression, using GAPDH as a reference. Relative expression was calculated by 2^–ΔΔCt (Pfaffl 2001). Real time PCR products were purified (Jena Bioscience, Germany), quantified (NanoDrop Q5000), and sequenced using an ABI 3730XL DNA Analyzer (Applied Biosystems). Forward and reverse sequences were aligned using Chromas 1.45 and BLAST tools (Altschul et al. 1990). SNPs were identified versus GenBank references, and amino acid sequences compared using MEGA6 (Tamura et al. 2007) (Table 2).

**Table 2:** Forward-reverse oligonucleotide-based real-time PCR primers for the immunological and antioxidant genes being studied

Investigated marker	Primer	Product size (bp)	Annealing Temperature (°C)	GenBank isolate	Origin
*IL-8*	F5′- ATGACTTCCAAGCTGGCTCTTG −3 R5′- GGATCTTGCTTTTCAGCTCTCT-3′	298	58	KF843702.1	
*IL-17*	F5′- GCGGCAGTGGTGAAGGCAGGA −3 R5′- TGACGCAGGTGCAGCCCACGGC −3′	385	60	XM_010992380.3	Present Research
*TLR4*	F5′- CACACCCACCTAGCTGTGGCTCT −3′ R5′- AAGTCCTCTAGAGATGCTAGGT - 3′	422	60	XM_010998333.3	
*TNFα*	F5′- CTCCTTGTCGCAGGAGCCACCA-3′ R5′- GATCTCAGCACTAAGTCGATCA - 3′	522	60	NM_001319880.1	
*PIN1*	F5′- CTGCCGCCCGGCTGGGAGAAG −3′ R5′- GCATCTTCAAATGGCTTCTGCAT- 3′	392	58	XM_031437325.2	
*IER3*	F5′- CTCCCGACCATGACCGTCCTGCG −3′ R5′- GAAGGCGGCCGGGTGTTGCTGGA −3′	444	60	XM_031435406.2	
* UBD*	F5′- GTGTGGTCCATTCTGAGCAATG-3′ R5′- GCCAATGCAGTGAGGTGTCAGA −3′	464	58	XM_010994241.3	
*TECPR1*	F5′- ACGCTCTCGTTGTCCATCACG −3′ R5′- ACAGGGCGAAGGAGTGCTTGGC - 3′	358	58	XM_074345688.1	
*PRDX2*	F5′- GAGCATGCTGAGGAGTTCCACA −3′ R5′- ACAGGGCGAAGGAGTGCTTGGC - 3′	417	58	XM_010993804.3	
*SOD3*	F5′- TGCTGGCGCTGCTCTGTGCCT −3′ R5′- GGCTGGTGACGTTGGGCTCGGT - 3′	339	60	XM_064487775.1	
*CAT*	F5′- CATCTAAAGGATCCAGATATGGT −3′ R5′- GTAATTAACTGGATTCCGGTT - 3′	453	58	XM_011000575.3	
*Nrf2*	F5′- CTAGATGAAGAGACAGGTGAAT −3′ R5′- ATGTGGACTACAGTTACCTACT - 3′	417	58	XM_031451712.2	
*GAPDH*	F5′- GGCGTGAACCACGAGAAGTA-3′ R5′- GGCGTGGACTGTGGTCATAA - 3′	141	60	XM_010990867.3	

IL-8= Interleukin 8; IL-17= Interleukin 8; TLR4= Toll-like receptor 4; TNFα = Tumor necrosis factor alpha; PIN1= Peptidylprolyl cis/trans isomerase, NIMA-interacting 1; IER3= Immediate early response 3; UBD= Ubiquitin D; TECPR1= Tectonin beta-propeller repeat containing 1; PRDX2= Peroxiredoxin 2; SOD3= Superoxide dismutase; CAT; Catalase; and Nrf2= Nuclear factor erythroid 2-related factor 2

### Biochemical analysis

Serum concentrations of glucose (mg/dl), total protein (g/dl), albumin (g/dl), urea (mg/dl), and creatinine (mg/dl) were determined using commercial diagnostic kits (Gamma Trade Company, Egypt). Calcium (mg/dl) levels were measured using kits supplied by BioMed, Egypt, while phosphors (mg/dl), magnesium (mg/dl), sodium (mmol/l), potassium (mmol/l), cloride (mmol/l) (TECO, USA; Spinreact, Spain). The activities of aspartate aminotransferase (AST) (U/L), alanine aminotransferase (ALT) (U/L) and alkaline phosphatase (ALP) (U/L) were analyzed using reagents provided by Spectrum Diagnostics (Egypt). Antioxidant biomarkers, including superoxide dismutase (SOD) (U/ml) and malondialdehyde (MDA) (nmol/ml), were quantified using assay kits obtained from Bio-Diagnostic, Egypt. All measurements were performed with a selective chemistry analyzer (Apple 302, USA). Serum globulin (g/dl) levels were calculated by subtracting albumin values from total protein concentrations (Fischbach and Dunning 2009).

### Statistical analysis

Independent Samples t-Test (SPSS software program, version 20, USA) was used to determine the differences in the biochemical parameters and gene expression of tested enzymes among animal of different disease status (diarrheic and non-diarrheic groups). This was utilized to assess whether or not the biochemical markers and SNPs found were significantly linked to the presence of diarrhea. The values were expressed as mean ± SD. Difference in the frequencies of each gene SNPs between diarrheic and healthy calves was statistically evaluated using Chi-square test to compare the distribution of the identified SNPs between the two groups using SPSS version 23, USA. A Linear Discriminant Analysis (LDA) was conducted to determine whether gene-level SNP averages could differentiate between diarrheic and healthy calves. The 12 gene average scores served as predictor variables, and the health status (diarrheic vs. healthy) was the grouping variable Statistical significance was set at *p* < 0.05.

## Results

### Clinical findings

The most common clinical findings in diarrheic calves based on clinical examination were anorexia, staggering movement, sunken eyes, diarrhea, dehydration, dullness, depression and pale mucous membranes. The feces were semi-fluid to watery in consistency and grayish to yellowish green in color, contained mucous, the perineum and tail were soiled with feces and mucous membranes were congested. There was a significant (*P* < 0.05) increase of body temperature, pulse and respiratory rates (40.7 ± 0.1 °C, 51.3 ± 1.2 Beats/min and 22.6 ± 0.8 Breaths/min), respectively in diarrheic calves in relation to control ones (38.4 ± 0.2 °C, 45.8 ± 0.6 Beats/min and 17.9 ± 0.5 Breaths/min), respectively Table 3.

**Table 3:** Changes in temperature, pulse and respiratory rates; in diarrheic Calve camel Calves compared to control ones (mean ± SE)

Parameters	Control calves	Diarrheic calves	*p*- value
Temperature (°C)	38.4 ± 0.2	40.7 ± 0.1*	0.001
Pulse rate (Beats/min)	45.8 ± 0.6	51.3 ± 1.2*	0.01
Respiratory rate (Breaths/min)	17.9 ± 0.5	22.6 ± 0.8*	0.01

(*) Statistically significant when *P* < 0.05

### Isolation, identification, and characterization of pathogens

Table 4 showed the intestinal bacterial isolation from both healthy and diarrheal camels. The data indicated that although *E. Coli*,* Salmonella*, and *C. perfringens* were identified from both groups, they were considerably more common in calves that had diarrhea (94.2%, 71.4%, and 82.9%, respectively). However, the prevalence of *Proteus species* was significantly higher in healthy calves (85.7%) compared to those who had diarrhea.

**Table 4:** The bacterial species isolated from healthy and diarrheic camels calves

Bacterium	Healthy % (*n*=35)	Diarrheic % (*n*=35)	Total % (*n*=70)
E. coli phoA	(18/35) 51.4%	(33/35) 94.2%	(51/70) 72.8%
Salmonella invA	(16/35) 45.7%	(25/35) 71.4%	(41/70) 58.5%
Proteus atpD	(30/35) 85.7%	(7/35) 20.0%	(37/70) 52.8%
C. perfringens alpha toxin	(13/35) 37.1%	(29/35) 82.9%	(42/70) 60%

**Fig. 1:**
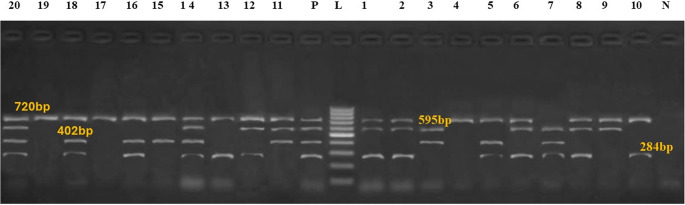
Multiplex PCR analysis of bacterial infection in fecal samples from camel with diarrhea and selected reference strains. Bands corresponding to *E. coli phoA* (720 bp), *Salmonella invA*(284 bp), *C. perfringens alpha toxin* (402 bp) and *Proteus atpD* (595 bp) are indicated. Lanes 1 and 20 are samples, L is 100-bp size ladder. Lanes 14 and 20 indicated mixed infection with all bacteria (*E. coli phoA*,* Salmonella invA*,* C. perfringens alpha toxin and Proteus atpD* respectively); lanes 3, 5, 9, 10, 12, 16 and infection within with *E. coli phoA*,* Salmonella invA and Proteus atpD* respectively); lane 1, and 13 mixed infection with *E. coli phoA and Salmonella invA* respectively. lane 2 mixed infection with *E. coli phoA and C. perfringens alpha toxin* respectively; lane 4 mixed infection with *Salmonella invA*,* C. perfringens alpha toxin and Proteus atpD* respectively); lane 6 mixed *E. coli phoA*,* Salmonella invA*,* and Proteus atpD* respectively); lane 7, 17 and 19 with *E. coli phoA* only, lane 8 mixed infection with *C. perfringens alpha toxin and Proteus atpD* respectively);,lane 11 mixed infection with *E. coli phoA*,* C. perfringens alpha toxin and Proteus atpD* respectively); lane 15 mixed infection with *E. coli phoA*,* and C. perfringens alpha toxin* respectively); lane 18 mixed infection with *E. coli phoA*,* salmonella invA*,* and C. perfringens alpha toxin* respectively)

In the multiplex PCR assay, primer pairs are crucial because they allow for simultaneous identification of the target strains, prevent cross-reactions, and guarantee the test’s sensitivity and specificity. By figuring out the ideal annealing temperature and adjusting the concentration of the primer pairs to guarantee equal amplification of each individual PCR result, we increased the multiplex PCR’s efficiency. The multiplex PCR experiment was exhibited in Fig. 1, where the bands at 720 bp, 284 bp, 402 bp, and 595 bp were distinct and intense. Products from *E. coli* (80%), *Salmonella spp*. (70%), *C. perfringens* alpha-toxin (60%), and *Proteus spp*. (42.8%) are shown in these bands, in that order. Table 5 showed the pathogen distribution in camel calves that had diarrhea. *E. Coli phoA* + *Proteus atpD* + *Salmonella invA* was the most prevalent pattern, occurring in 35% of samples. Just 15% of calves experienced diarrhea linked to *E. coli phoA*. *Salmonella invA* + *E. Coli phoA* (10%). As can be seen from the table, several calves had all four infections at the same time (10%; *C. perfringens + E. coli + Proteus + Salmonella*). *Proteus* was not the main pathogen in diarrheal calves, but it was a component of mixed infections. As a negative control in the multiplex PCR experiment, we substituted sterile Milli-Q water for template DNA. The most advantageous annealing temperature was 55 °C, which led to the amplification of equivalent amounts of the four target strains. This control did not display any PCR products.

**Table 5:** The distribution of pathogens found in diarrheal camel calves

Infection Pattern	Sample Count	Percentage of Samples
E. coli phoA + Proteus atpD + Salmonella invA	7	35%
E. coli phoA	3	15%
E. coli phoA + Salmonella invA	2	10%
C. perfringens alpha toxin + E. coli phoA	2	10%
C. perfringens alpha toxin + E. coli phoA + Proteus atpD + Salmonella invA	2	10%
C. perfringens alpha toxin + Proteus atpD + Salmonella invA	1	5%
C. perfringens alpha toxin + Proteus atpD	1	5%
C. perfringens alpha toxin + E. coli phoA + Proteus atpD	1	5%
C. perfringens alpha toxin + E. coli phoA + Salmonella invA	1	5%

### Virulence genes of *E. coli, Salmonella, C perfringens *and* Proteus*

**Fig. 2:**
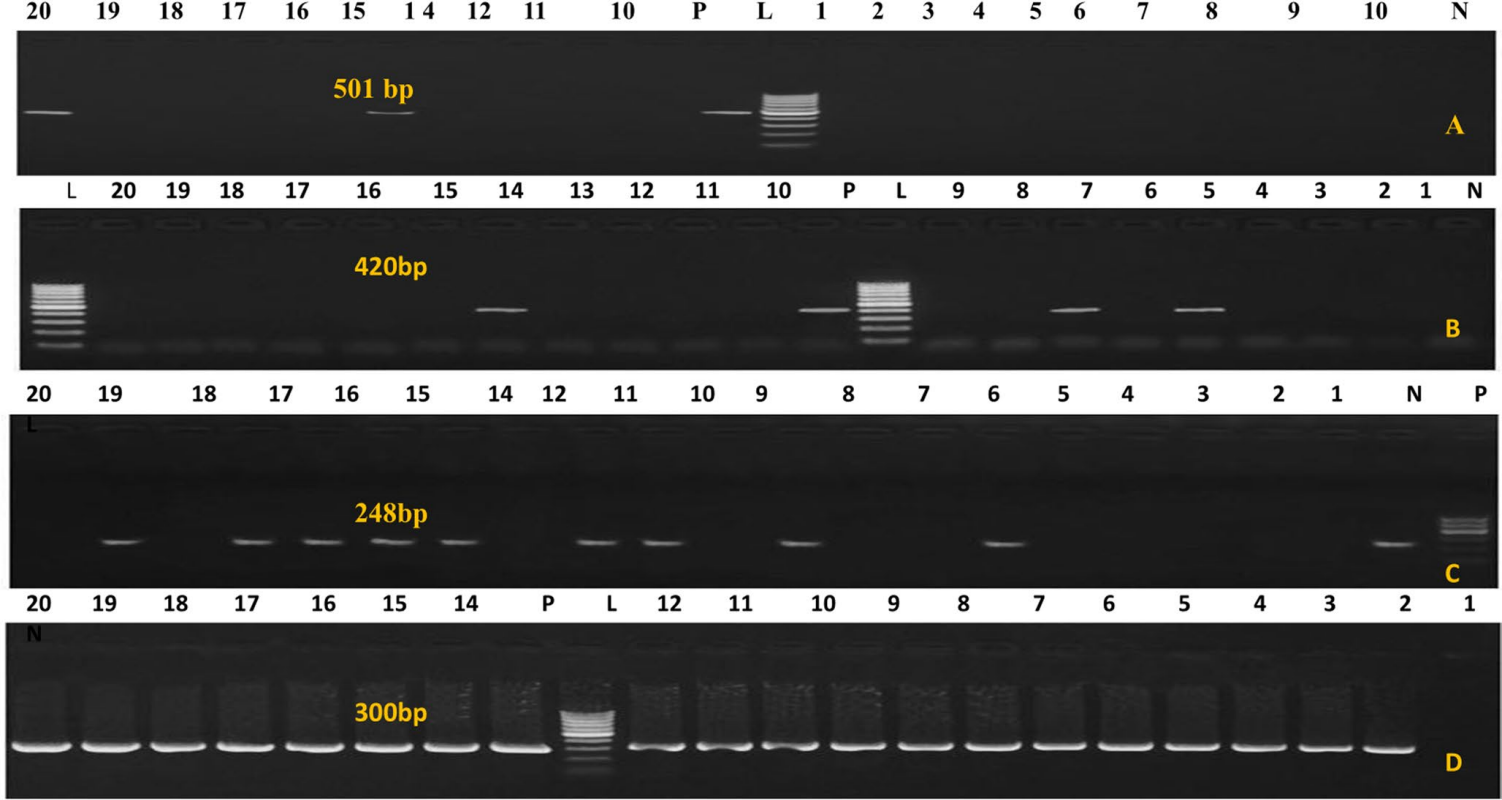
PCR amplification of some virulence gene of *E. coli. *(**A**)* papC* with indicted size 501bp; (**B**) *vat* gene with indicted size 420bp. (**C**) *eaeA* gene showed at 248 bp. (**D**) *iutA *gene is shown above all lanes represents the positive product at 300 bp. L is 100 bp ladder

**Fig. 3:**
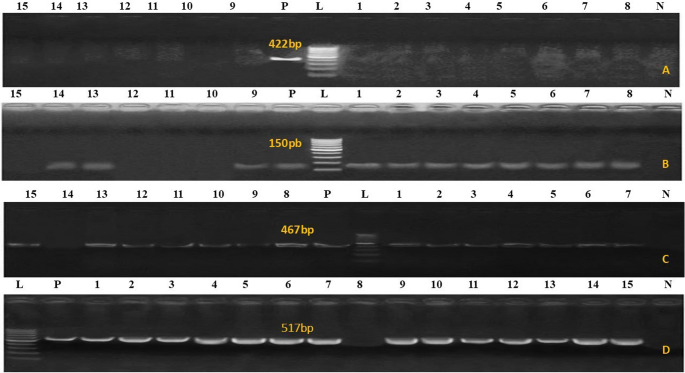
PCR amplification of *Salmonella virulence gene*. (**A**) *avrA* gene band was detected at 422 bp in control positive lane only. (**B**) *hilA* gene at 150 bp. (**C**) *bcfC* gene at 467 bp. (**D**) *sopB* gene at 517 bp M, Molecular weight marker showed 100–1000 bp DNA ladder

**Fig. 4:**
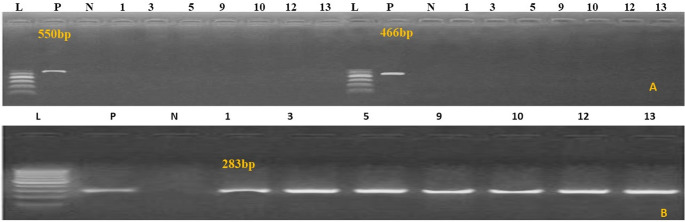
*C. perfringens* virulence genes. (**A**) *netB* gene 560 bp, *C. perfringens tpeL gene* 466 bp. (**B**) *plc* (α-toxin). DNA from different samples the type of code is shown above the all lanes represents the positive product of *C. perfringens* plc (α-toxin) at 283 bp. M, Molecular weight marker showed 100–1000 bp DNA ladder

**Fig. 5:**
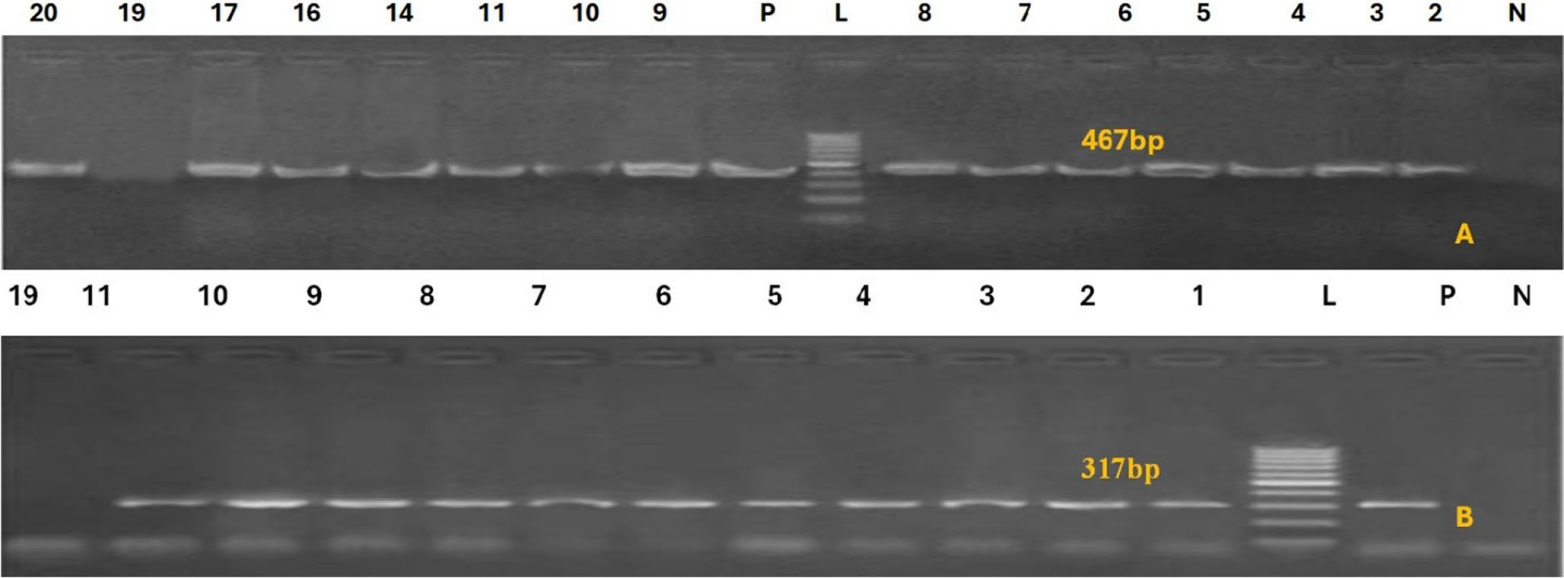
PCR amplification of *Proteus*spp. virulence genes (**A**) *rsbA*. is shown above the all lanes represents the positive product of *Proteus rsbA* at 467 bp, (**B**) *plc* (α-toxin) at 283 bp

Using primers listed in the materials and methods, the phoA gene was amplified to confirm the genetic identity of the *E. coli* isolates. The virulence genes papC (20%), vat (30%), eaeA (51%), and iutA (100%) were then detected by PCR in all verified isolates from diarrheal camel calves (Fig. 2A-D). As illustrated in Fig. 3A–D, PCR was used to screen all *salmonella* isolates from diarrheal camels for the presence or absence of certain virulence genes, such as avrA (0%), hilA (76%), bcfC (80%), and sopB (96%). As shown in Fig. 4A, B, PCR was used to test *C perfringens* positive isolates from diarrheal camels for the presence or lack of certain virulence genes, such as netB (0%), tpeL (0%), and plc (100%). PCR analysis was used to test *proteus* positive isolates from camels with diarrhea for the presence or absence of specific virulence genes, rsbA (60%) and ureC (91.4%), as shown in (Fig. 5A, B).

### Quantification, expression, and genetic variation of immunological and antioxidant genetic markers

**Fig. 6:**
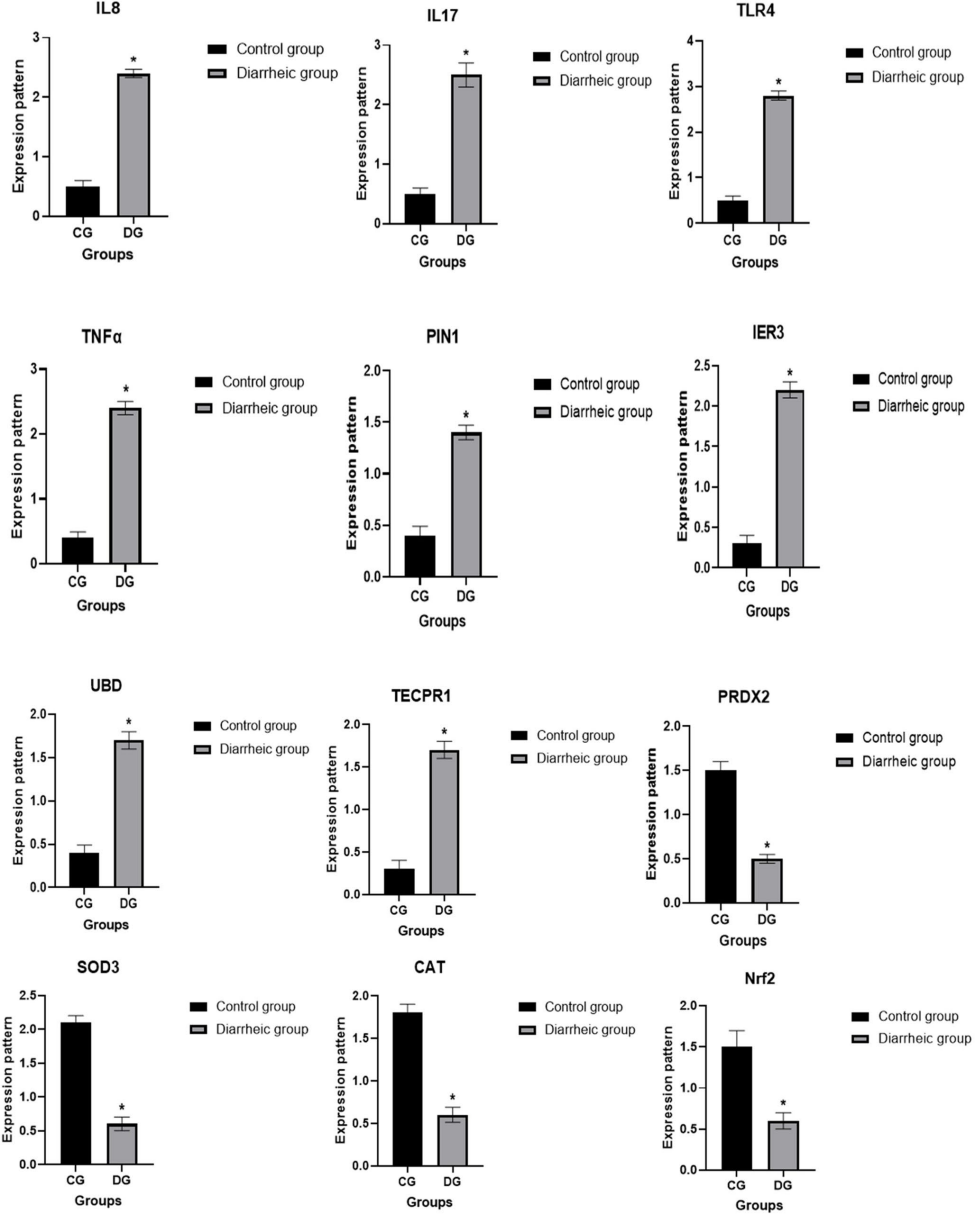
Healthy and diarrhea-affected camels have different (*IL-8*, *IL-17*, *TLR4*, *TNFα*, *PIN1*, *IER3*, *UBD*, *TECPR1*) and antioxidant (*PRDX2*, *SOD3*, *CAT*, *Nrf2*) genes transcript levels. When p is less than 0.05, significance is indicated by the symbol *

The transcript patterns for the evaluated immunological and antioxidant markers were shown in Fig. 6. The genes IL-8, IL-17, TLR4, TNFα, PIN1, IER3, UBD, and TECPR1 had significantly greater gene expression levels in diarrhea-affected calves compared to healthy ones. However, PRDX2, SOD3, CAT, and Nrf2 levels decreased. The highest possible level of mRNA for diarrheic camels was 2.85 ± 0.13 for TLR4, whereas the lowest amount of each gene was 0.53 ± 0.54 for PRDX2. Out of all the genes examined in the healthy camels, SOD3 had the highest possible level of mRNA (2.18 ± 0.13), whereas IER3 had the lowest amount (0.36 ± 0.12).

Using PCR-DNA sequence verdicts, IL-8 (298-bp), IL-17 (385-bp), TLR4 (422-bp), TNFα (522-bp), PIN1 (392-bp), IER3 (444-bp), UBD (464-bp), TECPR1 (358-bp), PRDX2 (417-bp), SOD3 (339-bp), CAT (453-bp), and Nrf2 (417-bp) genes were found to have different SNPs in the amplified DNA bases linked to diarrhea. Based on the DNA sequence variations between immunological and antioxidant indicators examined in the camels under study and the reference gene sequences retrieved from Gen Bank Figures (S1–S12), all the identified SNPs were approved.

The coding DNA sequences of the affected camels differed from those of the healthy ones due to the exonic region abnormalities that were seen in Table 6 in all of the immunological and antioxidant markers tested. Using DNA sequencing of immune and antioxidant genes, 22 SNPs were found; fourteen of them were non-synonymous and eight were synonymous.

**Table 6:** Diarrheic and healthy calves' immunological and antioxidant marker distributions with a single base differential and possible genetic change

Gene	SNPs	Healthy n = 35	Diarrhea n = 35	Total n = 70	Chi square value X2	p Value	kind of inherited change	Amino acid order and sort
*IL-8*	A145G	-/35	21/35	21/70	30	0.001	Non-synonymous	49 K to E
*IL-17*	T70G	-/35	18/35	18/70	24.2	0.001	Non-synonymous	24 F to V
	A171C	13/35	-/35	13/70	15.9	0.001	Synonymous	57 L
*TLR4*	T51C	28/35	-/35	28/70	46.6	0.001	Synonymous	17 C
	C219G	23/35	-/35	23/70	34.2	0.001	Non-synonymous	73 S to R
*TNFα*	A76C	17/35	-/35	17/70	22.4	0.001	Non-synonymous	26 M to L
	A366C	32/35	-/35	32/70	58.9	0.001	Synonymous	122 A
	A425G	-/35	25/35	25/70	38.8	0.001	Non-synonymous	142 D to G
*PIN1*	C237T	-/35	14/35	14/70	17.5	0.001	Synonymous	91 A
*IER3*	C47G	30/35	-/35	30/70	23.3	0.001	Non-synonymous	16 T to S
	G400T	-/35	13/35	13/70	15.9	0.001	Non-synonymous	134 A to S
*UBD*	G40A	19/35	-/35	19/70	26	0.001	Non-synonymous	14 S to N
	T74A	18/35	-/35	18/70	24.2	0.001	Non-synonymous	25 N to K
	C334A	-/35	14/35	14/70	17.5	0.001	Non-synonymous	112 A to E
*TECPR1*	T218C	21/35	-/35	21/70	30	0.001	Non-synonymous	73 M to T
*PRDX2*	G45T	-/35	15/35	15/70	19	0.001	Synonymous	15 G
	C198A	-/35	14/35	14/70	17.5	0.001	Synonymous	66 A
*SOD3*	T31C	24/35	-/35	24/70	36.5	0.001	Non-synonymous	11 M to T
*CAT*	G193A	13/35	-/35	13/70	15.9	0.001	Non-synonymous	65 D to N
*Nrf2*	T129C	-/35	29/35	29/70	25.9	0.001	Synonymous	43 P
	G172C	31/35	-/35	31/70	55.6	0.001	Non-synonymous	58 E to Q
	T363C	-/35	14/35	28/70	17.5	0.001	Synonymous	121Y

IL-8= Interleukin 8; IL-17= Interleukin 8; TLR4= Toll-like receptor 4; TNFα = Tumor necrosis factor alpha; PIN1= Peptidylprolyl cis/trans isomerase, NIMA-interacting 1; IER3= Immediate early response 3; UBD= Ubiquitin D; TECPR1= Tectonin beta-propeller repeat containing 1; PRDX2= Peroxiredoxin 2; SOD3= Superoxide dismutase; CAT; Catalase; and Nrf2= Nuclear factor erythroid 2-related factor 2. A= Alanine; C= Cysteine; D= Aspartic acid; E= Glutamic acid; F= Phenylalanine; G= Glycine; K= Lysine; L = Leucine; M= Methionine; N= Asparagine; P= Proline; Q= Glutamine; R= Argnine; S = Serine; T= Threonine; V = Valine; and Y= Tyrosine

A significant difference was detected in the frequencies of all examined gene SNPs among diarrheic and healthy calves (*p* < 0.005). Chi-square analysis was carried out for comparison of the distribution of all identified SNPs in all genes between diarrheic and healthy calves. The total chi-square value showed significant variation among the identified SNPs in all genes between resistant and affected animals (*p* < 0.05) (Table 6).

The discriminant analysis for the correlation between healthy status and gene types was displayed in Table 7. The organizational ramifications demonstrated that the model correctly identified either healthy or diarrheal calves in 100% of the situations. According to these findings, the SNP markers utilized in the model have a high degree of discriminatory power and could be helpful as prospective genetic markers for camel calves’ susceptibility to diarrhea.

**Table 7:** Discriminant analysis for classification of type of genes and healthy status of examined calves

			Predicted Group Membership		Total
			Healthy	Diseases	
	Count	Healthy	35	0	100
		Diseased	0	35	100
	%	Healthy	35	0.0	100.0
		Diseased	0.0	35	100.0

### Biochemical analysis

The present study showed significant (*P* > 0.05) high values of serum activity of AST, ALT, ALP and serum level of urea, creatinine, potassium and MDA with significant decrease in the serum values of glucose, total protein, Ca, P, Mg, Na, Cl and SOD in diarrheic calves compared with control healthy ones (Table 8).

**Table 8:** Biochemical parameters in control and diarrheic calve camels (mean ± SE)

Parameters	Control camels calves	Diarrheic camels calves	*p*-value	Reference value
Glucose (mg/dl)	160.6±3	105.6 ±1.7*	0.001	135 mg/dl (Faraz et al. 2021)
Total protein (g/dl)	5.2±0.2	4.2±0.1*	0.01	5.1 g/dl (Faraz et al. 2021)
Albumen (g/dl)	4.1±0.07	4.7± 0.2	0.05	4.4 g/dl (Islam et al. 2019)
Globulin (g/dl)	1±0.03	1±0.06	0.9	1.4 g/dl (Hafez et al. 2022)
Blood urea nitrogen (mg/dl)	45±0.5	55.6±1.7*	0.005	40 mg/dl (Faraz et al. 2021)
Creatinine (mg/dl)	0.6±0.005	0.7±0.03*	0.007	1 mg/dl (Ebissy et al. 2021)
AST (U/L)	37±1.1	68.3±1.2*	0.001	36.1 U/L (Kataria and Bhatia 1991)
ALT (U/L)	59.6±1.4	92.3±1.4*	0.001	46.8 U/L (Mousa et al. 2025)
ALP (U/L)	48.6±1.8	87±0.5*	0.001	49.5 U/L (Ebissy et al. 2021)
Calcium (mg/dl)	6.1±0.1	4.4±0.4*	0.03	8.5 mg/dl (Hafez 2006)
Phosphors (mg/dl)	4.5±0.1	3.4±0.1*	0.007	5.1 mg/dl (Hafez 2006)
Magnesium (mg/dl)	2.4±0.05	1.8±0.1*	0.01	2.3 mg/dl (Hafez 2006)
Sodium (mmol/l)	158±1	137.6±3.1*	0.006	139.7 mmol/l (Mousa et al. 2025)
Potassium (mmol/l)	1.8±0.07	2.8±0.05*	0.001	1.8 mmol/l (Hussein et al. 1992)
Cloride (mmol/l)	106±3.7	84.8±1.8*	0.007	110 mmol/l (Shoeib et al. 2019)
SOD (U/ml)	56±0.5	35±1.7*	0.001	50.6 U/ml (Ateya et al. 2021)
MDA (nmol/ml)	5.9±0.2	7±0.1*	0.02	2.7 nmol/ml (Darwish 2023)

*AST* Aspartate aminotransferase; *ALT* Alanine transaminase; *ALP* Alkaline phosphatase; *SOD* Super oxide dismutase; *MDA* Malondialdehyde. (*) Statistically significant when *P*< 0.05

## Discussion

Neonatal diarrhea remains a leading cause of morbidity and mortality in camel calves, mainly through dehydration, acidosis, and electrolyte imbalance (McClure 2001). The observed fever in diarrheic camel calves indicates systemic inflammation and infection (Risalde et al. 2011), consistent with earlier findings in diarrheic neonates of camels (Bessalah et al. 2016; Shahein et al. 2021) and other ruminants (El-Seadawy et al. 2020; Torche et al. 2020; Darwish et al. 2021; Hafez et al. 2022; Shehta et al. 2022).

The marked differences in bacterial isolation between healthy and diarrheic camel calves confirm the infectious nature of the disease. *E. coli* phoA was predominant in diarrheic calves (94.2%) versus healthy ones (51.4%), underscoring its central role in enteric disorders. Similar observations have been made in Egypt and Tunisia (Farag 2022; Tayh et al. 2022). The frequent detection of iutA (100%) and eaeA (51%) genes reflects strong colonization and iron-acquisition abilities, while papC (20%) and vat (30%) suggest additional adherence and virulence mechanisms (Mulvey 2002; Hussein et al. 2013). Theses finding were consistent with previous reports in camel neonates (Bessalah et al. 2016; Shahein et al. 2021).

*Salmonella* invA was also significantly more common in diarrheic camel calves (71.4%), supporting its role in camel enteritis (Shahein et al. 2021; Sallam et al. 2023). The high prevalence of invA, hilA, bcfC, and sopB genes indicates strong invasive and adhesive capacities, consistent with global findings (Liang et al. 2011). The absence of avrA suggests possible strain variation or geographic differences (Moustafa et al. 2020). Conversely, *Proteus* atpD was more frequent in healthy camel calves (85.7%), suggesting a commensal rather than pathogenic role, although ureC (91.4%) and rsbA (60%) indicate latent virulence potential (Ali and Yousif 2015; Algammal et al. 2021). *Clostridium perfringens* was isolated from 82.9% of diarrheic and 37.1% of healthy camel calves, highlighting its opportunistic behavior. Comparable findings were reported in diarrheic camel calves (Fayez et al. 2020). The consistent detection of plc confirms its pathogenic involvement, while the absence of netB and tpeL mirrors patterns in bovine enteric disease (Schlegel et al. 2012).

Mixed infections dominated diarrheic cases (85%), mainly *E. coli* phoA + *proteus* atpD + *salmonella* invA (35%), suggesting synergistic effects between bacterial adhesion, invasion, and toxin production. The frequent co-occurrence of *E. coli* with *salmonella* or *C. perfringens* likely enhances disease severity, while *proteus* appears as a secondary invader (Magaji et al. 2012; Algammal et al. 2021). In summary, neonatal diarrhea in camel calves is a multifactorial syndrome primarily involving *E. coli*,* Salmonella*, and *C. perfringens*, with *proteus* acting opportunistically. The predominance of mixed infections highlights the need for multiplex diagnostics, improved hygiene, and integrated control strategies to reduce disease incidence and losses in camel herds.

Gene expression analysis showed significant upregulation of immune-related genes (IL-8, IL-17, TLR4, TNFα, PIN1, IER3, UBD, TECPR1) and downregulation of antioxidant genes (PRDX2, SOD3, CAT, Nrf2) in diarrheic camel calves, indicating enhanced inflammation and oxidative stress. Similar expression profiles have been reported in diarrheic goats and lambs (Al-Sharif and Ateya 2023; Darwish et al. 2024), respectively. The genetic variation usually manifests first at the individual level and influences susceptibility to diarrheal diseases. Recurrent infectious disease outbreaks can promote natural selection at the population level over generations, increasing the frequency of protective genetic variations. Sequencing revealed nucleotide variations between diarrheic and healthy camel calves, suggesting SNP-related differences that may influence susceptibility to infection. Such associations between gene polymorphisms and diarrhea have been described in calves (Ateya et al. 2024), piglets (Yang et al. 2014; Cheng et al. 2021), goats (Al-Sharif and Ateya 2023) and Barki lambs (Darwish et al. 2024).

The upregulation of IL-8, IL-17, and TLR4 reflects activation of innate immune pathways (Ishigame et al. 2009; Botos et al. 2011; Al-Sharif and Ateya 2023), while PIN1, IER3, UBD, and TECPR1 are involved in apoptosis and autophagy (Hipp et al. 2005; Manganaro et al. 2010; Arlt and Schäfer 2011; Ogawa et al. 2011), supporting an infection-driven inflammatory response. The concurrent downregulation of antioxidant genes (Nrf2, PRDX2, SOD3, CAT) indicates impaired oxidative defense and increased cellular stress (Aslan et al. 2017). These alterations suggest that diarrhea in camel calves involves a disrupted balance between immune activation and antioxidant protection, consistent with pathogen-induced oxidative injury and barrier dysfunction (Pisani et al. 2009; Shi et al. 2019).

Diarrheic camel calves exhibited marked metabolic and biochemical disturbances compared with healthy controls. Hypoglycemia and hypoproteinemia were evident, in line with previous reports (Choi et al. 2021; Hafez et al. 2022), likely reflecting reduced intestinal absorption, luminal nutrient loss, and hepatic dysfunction induced by bacterial toxins (Constable et al. 2016). Significant elevations in serum creatinine, BUN, AST, ALT, and ALP suggest hepatic and renal stress, possibly resulting from dehydration, hypovolemia, and tissue injury, as previously noted in diarrheic ruminants (Ali et al. 2012; Ghanem et al. 2012; Askar et al. 2025).

Electrolyte profiles indicated decreased Ca, P, Mg, Na, and Cl and increased K, consistent with Hafez et al. (2022). These imbalances reflect intestinal loss and acidosis-induced shifts in ion distribution. Hypocalcemia, hypophosphatemia, and hypomagnesemia likely arise from malabsorption and fecal loss (Chernecky and Berger 2012; EL-dessouky and EL-Masry 2005), while hyperkalemia may result from acidosis and impaired renal excretion (Urgibl-Bauer et al. 2025). Oxidative stress was evident by elevated MDA and reduced SOD levels, consistent with enhanced lipid peroxidation and antioxidant depletion (Ahmed and Hassan 2007; Ghanem et al. 2012; Hafez et al. 2022). These findings underscore oxidative damage as a contributing factor in enteric pathology and suggest potential benefits of antioxidant supplementation in supportive therapy.

Future research should take into account the limitation that the current study revealed. The identity of a species cannot be conclusively confirmed by PCR detection of virulence genes alone. Phylogenetic analysis or PCR product sequencing would, in fact, offer more convincing proof. Therefore, in order to strengthen species-level identification, prepare to confirm the identity of these isolates using whole-genome analysis or 16 S rRNA gene sequencing.

## Conclusions

This study identified diverse bacterial pathogens in diarrheic camel calves in Egypt and the identified SNPs in immune (IL-8, IL-17, TLR4, TNFα, PIN1, IER3, UBD, TECPR1) and antioxidant (PRDX2, SOD3, CAT, Nrf2) genes were significantly associated with the occurrence of diarrhea, suggesting that these polymorphisms may influence host susceptibility through altered cytokine expression or oxidative defense mechanisms. Distinct gene expression patterns between healthy and diarrheic calves, along with biochemical and antioxidant alterations, highlight opportunities for health monitoring and selective breeding to enhance natural resistance.

## Supplementary Information

Below is the link to the electronic supplementary material.

Supplementary Material 1

## Data Availability

The data will be available from the corresponding author upon request.
