# Supplementary material for: Integrative analysis of genetic, biochemical, and microbial factors in camel calf diarrhea
Source: Vet Res Commun. 2026 Feb 6;50(2):147. doi: 10.1007/s11259-025-11027-w (PMC12881016; doi:10.1007/s11259-025-11027-w)
Supplement: Supplementary file 1 — Supplementary Material 1 [file 11259_2025_11027_MOESM1_ESM.pdf]

```
D               ATGACTTCCAAGCTGGCTCTTGTGGCCAGCTTTCTTGATTTCTGCAGCTTGTGTGAAGCT       60
KF843702.1      ATGACTTCCAAGCTGGCTCTTGTGGCCAGCTTTCTTGATTTCTGCAGCTTGTGTGAAGCT       60
H               ATGACTTCCAAGCTGGCTCTTGTGGCCAGCTTTCTTGATTTCTGCAGCTTGTGTGAAGCT       60
                ************************************************************

D               GCAGTTCTGTCAAGACTGAGTTCAGAACTTCGATGCCAGTGCATAAATACACACTCCACA       120
KF843702.1      GCAGTTCTGTCAAGACTGAGTTCAGAACTTCGATGCCAGTGCATAAATACACACTCCACA       120
H               GCAGTTCTGTCAAGACTGAGTTCAGAACTTCGATGCCAGTGCATAAATACACACTCCACA       120
                ************************************************************

D               CCTTTCCACCCCAAATTTATCAAAGAACTAAGAGTGATTGAGAGTGGACCTCACTGTGAA       180
KF843702.1      CCTTTCCACCCCAAATTTATCAAAAAACTAAGAGTGATTGAGAGTGGACCTCACTGTGAA       180
H               CCTTTCCACCCCAAATTTATCAAAAAACTAAGAGTGATTGAGAGTGGACCTCACTGTGAA       180
                ************************ ***********************************

D               AATACAAAAATCATTGTTAAGCTTGCCAATGGAAGAGAGGTCTGCCTGAATCCCAAGGAA       240
KF843702.1      AATACAAAAATCATTGTTAAGCTTGCCAATGGAAGAGAGGTCTGCCTGAATCCCAAGGAA       240
H               AATACAAAAATCATTGTTAAGCTTGCCAATGGAAGAGAGGTCTGCCTGAATCCCAAGGAA       240
                ************************************************************

D               AAGTGGGTGCAAAAGGTTGTGGAGATTTTTTTGAAGAGAGCTGAAAAGCAAGATCCAT298
KF843702.1      AAGTGGGTGCAAAAGGTTGTGGAGATTTTTTTGAAGAGAGCTGAAAAGCAAGATCC--296
H               AAGTGGGTGCAAAAGGTTGTGGAGATTTTTTTGAAGAGAGCTGAAAAGCAAGATCCAT298
                ********************************************************
```

Figure S1. An illustration showing how the IL-8 gene (298 bp) aligns in healthy calves (H) and those with diarrhea (D).

```
XM_010992380.3      GCGGCAGTGGTGAAGGCAGGAGCCATAATCCCACAGAGCCCAGGATGCCCGATTACCGAG   60
H                   GCGGCAGTGGTGAAGGCAGGAGCCATAATCCCACAGAGCCCAGGATGCCCGATTACCGAG   60
D                   GCGGCAGTGGTGAAGGCAGGAGCCATAATCCCACAGAGCCCAGGATGCCCGATTACCGAG   60
                    ************************************************************

XM_010992380.3      GACAAGAAATTCCCTCAGCACGTGAAGCTCAACTTGAACATCCTTAACCGGAACACGAAT   120
H                   GACAAGAAATTCCCTCAGCACGTGAAGCTCAACTTGAACATCCTTAACCGGAACACGAAT   120
D                   GACAAGAAAGTCCCTCAGCACGTGAAGCTCAACTTGAACATCCTTAACCGGAACACGAAT   120
                    ********* **************************************************

XM_010992380.3      CCCAAAAGGCCCACCGATTACTATAAACGGTCCACCTCACCTTGGACTCTACACCGCAAC   180
H                   CCCAAAAGGCCCACCGATTACTATAAACGGTCCACCTCACCTTGGACTCTCCACCGCAAC   180
D                   CCCAAAAGGCCCACCGATTACTATAAACGGTCCACCTCACCTTGGACTCTACACCGCAAC   180
                    ************************************************** *********

XM_010992380.3      GAGGACCCCGAGCGGTACCCCTCTGTGATCTGGGAGGCCAAGTGCAGCCACTCGGGCTGT   240
H                   GAGGACCCCGAGCGGTACCCCTCTGTGATCTGGGAGGCCAAGTGCAGCCACTCGGGCTGT   240
D                   GAGGACCCCGAGCGGTACCCCTCTGTGATCTGGGAGGCCAAGTGCAGCCACTCGGGCTGT   240
                    ************************************************************

XM_010992380.3      GTCAACGCTAAGGGGAAGGTCGACCACCACATGAACTCCGTCCCCATCCAGCAAGAGATC   300
H                   GTCAACGCTAAGGGGAAGGTCGACCACCACATGAACTCCGTCCCCATCCAGCAAGAGATC   300
D                   GTCAACGCTAAGGGGAAGGTCGACCACCACATGAACTCCGTCCCCATCCAGCAAGAGATC   300
                    ************************************************************

XM_010992380.3      CTGGTCCTGCGACGGGAGCCTCAGCACTGCCCACACTCCTTCCGGCTGGAGAAGATGCTG   360
H                   CTGGTCCTGCGACGGGAGCCTCAGCACTGCCCACACTCCTTCCGGCTGGAGAAGATGCTG   360
D                   CTGGTCCTGCGACGGGAGCCTCAGCACTGCCCACACTCCTTCCGGCTGGAGAAGATGCTG   360
                    ************************************************************

XM_010992380.3      GTGGCCGTGGGCTGCACCTGCGTCA 385
H                   GTGGCCGTGGGCTGCACCTGCGTCA 385
D                   GTGGCCGTGGGCTGCACCTGCGTCA 385
                    *************************
```

Figure S2. An illustration showing how the IL-17 gene (385 bp) aligns in healthy calves (H) and those with diarrhea (D).

```
XM_010998333.3      CACACCCACCTAGCTGTGGCTCTGATGACAGCCATGGCCTTTCTCTCCTGTCTGAGATCC    60
H                   CACACCCACCTAGCTGTGGCTCTGATGACAGCCATGGCCTTTCTCTCCTGCCTGAGATCC    60
D                   CACACCCACCTAGCTGTGGCTCTGATGACAGCCATGGCCTTTCTCTCCTGTCTGAGATCC    60
                    ************************************************** *********

XM_010998333.3      GAGAGCTGGGAACCCTGTGTGCAGGTGGTTCCTAACATTAGTTACCAATGCATGGAGCTG    120
H                   GAGAGCTGGGAACCCTGTGTGCAGGTGGTTCCTAACATTAGTTACCAATGCATGGAGCTG    120
D                   GAGAGCTGGGAACCCTGTGTGCAGGTGGTTCCTAACATTAGTTACCAATGCATGGAGCTG    120
                    ************************************************************

XM_010998333.3      AATCTCTACAAAGTCCCAGACAACATCCCCACATCGACCAAGATACTGGATCTCAGCTTT    180
H                   AATCTCTACAAAGTCCCAGACAACATCCCCACATCGACCAAGATACTGGATCTCAGCTTT    180
D                   AATCTCTACAAAGTCCCAGACAACATCCCCACATCGACCAAGATACTGGATCTCAGCTTT    180
                    ************************************************************

XM_010998333.3      AACCACCTGAATCATTTAGGCAGCCATAGCTTCTCCAGCTTCCCAGAACTGCAGGTGCTG    240
H                   AACCACCTGAATCATTTAGGCAGCCATAGCTTCTCCAGGTTCCCAGAACTGCAGGTGCTG    240
D                   AACCACCTGAATCATTTAGGCAGCCATAGCTTCTCCAGCTTCCCAGAACTGCAGGTGCTG    240
                    ************************************** *********************

XM_010998333.3      GATTTATCCAGATGTGAAATTCAGATGATTGACGATAATGCGTATCAGGGGCTAAACCAC    300
H                   GATTTATCCAGATGTGAAATTCAGATGATTGACGATAATGCGTATCAGGGGCTAAACCAC    300
D                   GATTTATCCAGATGTGAAATTCAGATGATTGACGATAATGCGTATCAGGGGCTAAACCAC    300
                    ************************************************************

XM_010998333.3      CTTGCCACCTTGATATTGACGGGAAACCCTATCCAGAGTTTAGCCCTGAGAGCCTTTTCT    360
H                   CTTGCCACCTTGATATTGACGGGAAACCCTATCCAGAGTTTAGCCCTGAGAGCCTTTTCT    360
D                   CTTGCCACCTTGATATTGACGGGAAACCCTATCCAGAGTTTAGCCCTGAGAGCCTTTTCT    360
                    ************************************************************

XM_010998333.3      GGAATACCGAGTTTACAGAAGCTGGTTGCTGTGGAGACAAACCTAGCATCTCTAGAGGAC    420
H                   GGAATACCGAGTTTACAGAAGCTGGTTGCTGTGGAGACAAACCTAGCATCTCTAGAGGAC    420
D                   GGAATACCGAGTTTACAGAAGCTGGTTGCTGTGGAGACAAACCTAGCATCTCTAGAGGAC    420
                    ************************************************************

XM_010998333.3      TT      422
H                   TT      422
D                   TT      422
                    **
```

Figure S3. An illustration showing how the TLR4 gene (422 bp) aligns in healthy calves (H) and those with diarrhea (D).

```
NM_001319880.1      CTCCTTGTCGCAGGAGCCACCACGCTCTTTTGCCTGCTGCACTTTGGGGTAATTGGGCCC   60
H                   CTCCTTGTCGCAGGAGCCACCACGCTCTTTTGCCTGCTGCACTTTGGGGTAATTGGGCCC   60
D                   CTCCTTGTCGCAGGAGCCACCACGCTCTTTTGCCTGCTGCACTTTGGGGTAATTGGGCCC   60
                    ************************************************************

NM_001319880.1      CAGAAAGAAGAGCTCATGACTGGCCTCCAGCTCATGAACCCTCTGGCCCAGACCCTCAGA   120
H                   CAGAAAGAAGAGCTCCTGACTGGCCTCCAGCTCATGAACCCTCTGGCCCAGACCCTCAGA   120
D                   CAGAAAGAAGAGCTCATGACTGGCCTCCAGCTCATGAACCCTCTGGCCCAGACCCTCAGA   120
                    *************** ********************************************

NM_001319880.1      TCATCTTCTCAAGCCTCAAGAGATAAGCCTGTCGCCCATGTTGTAGCCGACCCCGCTGCC   180
H                   TCATCTTCTCAAGCCTCAAGAGATAAGCCTGTCGCCCATGTTGTAGCCGACCCCGCTGCC   180
D                   TCATCTTCTCAAGCCTCAAGAGATAAGCCTGTCGCCCATGTTGTAGCCGACCCCGCTGCC   180
                    ************************************************************

NM_001319880.1      CAGGGGCAGCTGCAGTGGGAGAAGCGGTTTGCCAATACCCTCCTGGCCAATGGTGTGAAG   240
H                   CAGGGGCAGCTGCAGTGGGAGAAGCGGTTTGCCAATACCCTCCTGGCCAATGGTGTGAAG   240
D                   CAGGGGCAGCTGCAGTGGGAGAAGCGGTTTGCCAATACCCTCCTGGCCAATGGTGTGAAG   240
                    ************************************************************

NM_001319880.1      CTGGAAGACAACCAGCTGGTGGTACCGACTGATGGGCTATACCTCATCTACTCCCAGGTC   300
H                   CTGGAAGACAACCAGCTGGTGGTACCGACTGATGGGCTATACCTCATCTACTCCCAGGTC   300
D                   CTGGAAGACAACCAGCTGGTGGTACCGACTGATGGGCTATACCTCATCTACTCCCAGGTC   300
                    ************************************************************

NM_001319880.1      CTCTTCAGTGGCCAGCGCTGCCCCTCCACCCCTGTGTTCCTCACTCACACCATCAGCCGC   360
H                   CTCTTCAGTGGCCAGCGCTGCCCCTCCACCCCTGTGTTCCTCACTCACACCATCAGCCGC   360
D                   CTCTTCAGTGGCCAGCGCTGCCCCTCCACCCCTGTGTTCCTCACTCACACCATCAGCCGC   360
                    ************************************************************

NM_001319880.1      CTTGCAGTCTCCTACCCAAACAAAGCCAACCTCCTCTCTGCCATCAAGAGCCCTTGCCAG   420
H                   CTTGCCGTCTCCTACCCAAACAAAGCCAACCTCCTCTCTGCCATCAAGAGCCCTTGCCAG   420
D                   CTTGCAGTCTCCTACCCAAACAAAGCCAACCTCCTCTCTGCCATCAAGAGCCCTTGCCAG   420
                    ***** ******************************************************

NM_001319880.1      GGGGACACCTCAGAGGAGGCTGAGGCCAAGCCCTGGTACGAGCCCATCTATCTGGGAGGG   480
H                   GGGGACACCTCAGAGGAGGCTGAGGCCAAGCCCTGGTACGAGCCCATCTATCTGGGAGGG   480
D                   GGGGGCACCTCAGAGGAGGCTGAGGCCAAGCCCTGGTACGAGCCCATCTATCTGGGAGGG   480
                    **** *******************************************************

NM_001319880.1      GTCTTCCAGCTGGAGAAGGATGATCGACTTAGTGCTGAGATC   522
H                   GTCTTCCAGCTGGAGAAGGATGATCGACTTAGTGCTGAGATC   522
D                   GTCTTCCAGCTGGAGAAGGATGATCGACTTAGTGCTGAGATC   522
                    ******************************************
```

Figure S4. An illustration showing how the TNFα gene (522 bp) aligns in healthy calves (H) and those with diarrhea (D).

```
XM_031437325.2      CTGCCGCCCGGCTGGGAGAAGCGCATGAGCCGCAGCTCAGGCCGGGTGTACTACTTCAAT   60
H                   CTGCCGCCCGGCTGGGAGAAGCGCATGAGCCGCAGCTCAGGCCGGGTGTACTACTTCAAT   60
D                   CTGCCGCCCGGCTGGGAGAAGCGCATGAGCCGCAGCTCAGGCCGGGTGTACTACTTCAAT   60
                    ************************************************************

XM_031437325.2      CACATCACTAACGCCAGCCAGTGGGAGCGGCCAAGTGGCAACAGCAGTGGCGGCGGCAAA   120
H                   CACATCACTAACGCCAGCCAGTGGGAGCGGCCAAGTGGCAACAGCAGTGGCGGCGGCAAA   120
D                   CACATCACTAACGCCAGCCAGTGGGAGCGGCCAAGTGGCAACAGCAGTGGCGGCGGCAAA   120
                    ************************************************************

XM_031437325.2      AATGGCCAAGGGGAGCCCACCAGGGTCCGCTGCTCACACCTGCTAGTCAAGCACAGCCAG   180
H                   AATGGCCAAGGGGAGCCCACCAGGGTCCGCTGCTCACACCTGCTAGTCAAGCACAGCCAG   180
D                   AATGGCCAAGGGGAGCCCACCAGGGTCCGCTGCTCACACCTGCTAGTCAAGCACAGCCAG   180
                    ************************************************************

XM_031437325.2      TCAAGGCGGCCCTCGTCCTGGCGACAGGAGAAGATCACCCGGACCAAGGAGGAGGCCCTG   240
H                   TCAAGGCGGCCCTCGTCCTGGCGACAGGAGAAGATCACCCGGACCAAGGAGGAGGCCCTG   240
D                   TCAAGGCGGCCCTCGTCCTGGCGACAGGAGAAGATCACCCGGACCAAGGAGGAGGCTCTA   240
                    ******************************************************** **

XM_031437325.2      GAGCTGATCAACGGCTACATCCAGAAGATCAAGTCGGGAGAAGAGGACTTTGAGTCTCTG   300
H                   GAGCTGATCAACGGCTACATCCAGAAGATCAAGTCGGGAGAAGAGGACTTTGAGTCTCTG   300
D                   GAGCTGATCAACGGCTACATCCAGAAGATCAAGTCGGGAGAAGAGGACTTTGAGTCTCTG   300
                    ************************************************************

XM_031437325.2      GCCTCACAGTTCAGCGACTGCAGCTCCGCCAAGGCCAGGGGAGACCTGGGTGCCTTCAGC   360
H                   GCCTCACAGTTCAGCGACTGCAGCTCCGCCAAGGCCAGGGGAGACCTGGGTGCCTTCAGC   360
D                   GCCTCACAGTTCAGCGACTGCAGCTCCGCCAAGGCCAGGGGAGACCTGGGTGCCTTCAGC   360
                    ************************************************************

XM_031437325.2      AGAGGTCAGATGCAGAAGCCATTTGAAGATGC   392
H                   AGAGGTCAGATGCAGAAGCCATTTGAAGATGC   392
D                   AGAGGTCAGATGCAGAAGCCATTTGAAGATGC   392
                    ********************************
```

Figure S5. An illustration showing how the PIN1 gene (522 bp) aligns in healthy calves (H) and those with diarrhea (D).

```
XM_031435406.2      CTCCCGACCATGACCGTCCTGCGGGCCCCGACCCCGGTCCCCTCCACCAGCCCGGGACCC   60
H                   CTCCCGACCATGACCGTCCTGCGGGCCCCGACCCCGGTCCCCTCCAGCAGCCCGGGACCC   60
D                   CTCCCGACCATGACCGTCCTGCGGGCCCCGACCCCGGTCCCCTCCACCAGCCCGGGACCC   60
                    ********************************************** *************

XM_031435406.2      CGGAGGGGCTCCGGTCCTGAGATCTTCACCTTCGACCCTCTCCCAGAGCCCGCGGTGGCA   120
H                   CGGAGGGGCTCCGGTCCTGAGATCTTCACCTTCGACCCTCTCCCAGAGCCCGCGGTGGCA   120
D                   CGGAGGGGCTCCGGTCCTGAGATCTTCACCTTCGACCCTCTCCCAGAGCCCGCGGTGGCA   120
                    ************************************************************

XM_031435406.2      CCCGCTGCGCGCCCCAGCGCCTCCCGCGGGCACCGAAAGCGCAGCCGTAGGGTCCTGTAC   180
H                   CCCGCTGCGCGCCCCAGCGCCTCCCGCGGGCACCGAAAGCGCAGCCGTAGGGTCCTGTAC   180
D                   CCCGCTGCGCGCCCCAGCGCCTCCCGCGGGCACCGAAAGCGCAGCCGTAGGGTCCTGTAC   180
                    ************************************************************

XM_031435406.2      CCACGAGTGGTCCGGCGCCAGCTGCCAGTCGAGGATCCGAACCCTGCCAAAAGGCTGCTC   240
H                   CCACGAGTGGTCCGGCGCCAGCTGCCAGTCGAGGATCCGAACCCTGCCAAAAGGCTGCTC   240
D                   CCACGAGTGGTCCGGCGCCAGCTGCCAGTCGAGGATCCGAACCCTGCCAAAAGGCTGCTC   240
                    ************************************************************

XM_031435406.2      TTTCTCCTGCTCACCATCATCTTCTGCCAGATCCTGACGGCTGAAGAGAGTGTGTCGACA   300
H                   TTTCTCCTGCTCACCATCATCTTCTGCCAGATCCTGACGGCTGAAGAGAGTGTGTCGACA   300
D                   TTTCTCCTGCTCACCATCATCTTCTGCCAGATCCTGACGGCTGAAGAGAGTGTGTCGACA   300
                    ************************************************************

XM_031435406.2      CCCCTGGCCCAGGAGGACACCCCCAGCGCCCCGTCCCCCGCACCCACAGCTGCGCCCCCG   360
H                   CCCCTGGCCCAGGAGGACACCCCCAGCGCCCCGTCCCCCGCACCCACAGCTGCGCCCCCG   360
D                   CCCCTGGCCCAGGAGGACACCCCCAGCGCCCCGTCCCCCGCACCCACAGCTGCGCCCCCG   360
                    ************************************************************

XM_031435406.2      GTCGAGCCCCTTAATCTGACCTCGGAGCCCTCAGACTACGCTTTGGACCTCAGCACTTTT   420
H                   GTCGAGCCCCTTAATCTGACCTCGGAGCCCTCAGACTACGCTTTGGACCTCAGCACTTTT   420
D                   GTCGAGCCCCTTAATCTGACCTCGGAGCCCTCAGACTACTCTTTGGACCTCAGCACTTTT   420
                    *************************************** ********************

XM_031435406.2      CTCCAGCAACACCCGGCCGCCTTC  444
H                   CTCCAGCAACACCCGGCCGCCTTC  444
D                   CTCCAGCAACACCCGGCCGCCTTC  444
                    ************************
```

Figure S6. An illustration showing how the IER3 gene (444 bp) aligns in healthy calves (H) and those with diarrhea (D).

```
XM_010994241.3      GTGTGGTCCATTCTGAGCAATGGAATTCAATGACTTTCAGTGCCAACTTGGAAGACAGAG   60
H                   GTGTGGTCCATTCTGAGCAATGGAATTCAATGACTTTCAATGCCAACTTGGAAGACAGAG   60
D                   GTGTGGTCCATTCTGAGCAATGGAATTCAATGACTTTCAGTGCCAACTTGGAAGACAGAG   60
                    *************************************** ********************

XM_010994241.3      TGAAGAAGATCAATGAACATGTCCGGTCTAAGACCAAGATTCCCGTGCAGGAGCAGGTCC   120
H                   TGAAGAAGATCAAAGAACATGTCCGGTCTAAGACCAAGATTCCCGTGCAGGAGCAGGTCC   120
D                   TGAAGAAGATCAATGAACATGTCCGGTCTAAGACCAAGATTCCCGTGCAGGAGCAGGTCC   120
                    ************* **********************************************

XM_010994241.3      TCCAGCTGGGCTCGAAGACCCTAAAGCCCCAGAGAACCCTGTCATCTTATGGTATCGACA   180
H                   TCCAGCTGGGCTCGAAGACCCTAAAGCCCCAGAGAACCCTGTCATCTTATGGTATCGACA   180
D                   TCCAGCTGGGCTCGAAGACCCTAAAGCCCCAGAGAACCCTGTCATCTTATGGTATCGACA   180
                    ************************************************************

XM_010994241.3      AGGAGACGACAATCCACCTCACCCTGAAGGTGGTGAAGCCCAGTGATGAGGAGCTGCCAT   240
H                   AGGAGACGACAATCCACCTCACCCTGAAGGTGGTGAAGCCCAGTGATGAGGAGCTGCCAT   240
D                   AGGAGACGACAATCCACCTCACCCTGAAGGTGGTGAAGCCCAGTGATGAGGAGCTGCCAT   240
                    ************************************************************

XM_010994241.3      TGGTTTTGGTGGAGTCAGGTGCTGAGGGGCAGAGGCACCTCCTCCAGGTGCGGAGGTCCA   300
H                   TGGTTTTGGTGGAGTCAGGTGCTGAGGGGCAGAGGCACCTCCTCCAGGTGCGGAGGTCCA   300
D                   TGGTTTTGGTGGAGTCAGGTGCTGAGGGGCAGAGGCACCTCCTCCAGGTGCGGAGGTCCA   300
                    ************************************************************

XM_010994241.3      GCTCAGTGGCCCAGGTGAAGGAGATGATTAAGGCGAAGACCGCTATAATTCCTAAGAAGC   360
H                   GCTCAGTGGCCCAGGTGAAGGAGATGATTAAGGCGAAGACCGCTATAATTCCTAAGAAGC   360
D                   GCTCAGTGGCCCAGGTGAAGGAGATGATTAAGGAGAAGACCGCTATAATTCCTAAGAAGC   360
                    ********************************* **************************

XM_010994241.3      AGATTGTGACTTGCAATGGAAAAAGATTGGAAGATGGGAAGATCCTGGGAGATTATGGCA   420
H                   AGATTGTGACTTGCAATGGAAAAAGATTGGAAGATGGGAAGATCCTGGGAGATTATGGCA   420
D                   AGATTGTGACTTGCAATGGAAAAAGATTGGAAGATGGGAAGATCCTGGGAGATTATGGCA   420
                    ************************************************************

XM_010994241.3      TCAGAAAGGGCAATATACTCTTTCTGACACCTCACTGCATTGGC 464
H                   TCAGAAAGGGCAATATACTCTTTCTGACACCTCACTGCATTGGC 464
D                   TCAGAAAGGGCAATATACTCTTTCTGACACCTCACTGCATTGGC 464
                    ********************************************
```

Figure S7. An illustration showing how the UBD gene (464 bp) aligns in healthy calves (H) and those with diarrhea (D).

```
XM_074345688.1      ACGCTCTCGTTGTCCATCACGCCTGCCCAAACCGCCGCCTGGCGGAAGCAGATCTTCCAG   60
H                   ACGCTCTCGTTGTCCATCACGCCTGCCCAAACCGCCGCCTGGCGGAAGCAGATCTTCCAG   60
D                   ACGCTCTCGTTGTCCATCACGCCTGCCCAAACCGCCGCCTGGCGGAAGCAGATCTTCCAG   60
                    ************************************************************

XM_074345688.1      CAGCTCACGGAAAGGACCAAGCGCGAGCTGGAGAACTTCCGACACTATGAGCAGGCCGTG   120
H                   CAGCTCACGGAAAGGACCAAGCGCGAGCTGGAGAACTTCCGACACTATGAGCAGGCCGTG   120
D                   CAGCTCACGGAAAGGACCAAGCGCGAGCTGGAGAACTTCCGACACTATGAGCAGGCCGTG   120
                    ************************************************************

XM_074345688.1      GAGCAGTCGGTGTGGGTGAAGACGGGGGCCCTGCAGTGGTGGTGTGACTGGAAACCGCAC   180
H                   GAGCAGTCGGTGTGGGTGAAGACGGGGGCCCTGCAGTGGTGGTGTGACTGGAAACCGCAC   180
D                   GAGCAGTCGGTGTGGGTGAAGACGGGGGCCCTGCAGTGGTGGTGTGACTGGAAACCGCAC   180
                    ************************************************************

XM_074345688.1      AAGTGGGTGGACGTCCGAGTGGCCCTGGAGCAGTTCATGGGGCACGACGGGGCTCGGGAC   240
H                   AAGTGGGTGGACGTCCGAGTGGCCCTGGAGCAGTTCACGGGGCACGACGGGGCTCGGGAC   240
D                   AAGTGGGTGGACGTCCGAGTGGCCCTGGAGCAGTTCATGGGGCACGACGGGGCTCGGGAC   240
                    ************************************* **********************

XM_074345688.1      AGCATCCTCTTCATCTACTACGCGGTCCACGAGGAGAAGAAGTATGTCCACGTGTTCCTC   300
H                   AGCATCCTCTTCATCTACTACGCGGTCCACGAGGAGAAGAAGTATGTCCACGTGTTCCTC   300
D                   AGCATCCTCTTCATCTACTACGCGGTCCACGAGGAGAAGAAGTATGTCCACGTGTTCCTC   300
                    ************************************************************

XM_074345688.1      AACGAGGTCACGGCGCTGGTCCCCATGCTGCACGAGGCCAAGCACTCCTTCGCCCTGT     358
H                   AACGAGGTCACGGCGCTGGTCCCCATGCTGCACGAGGCCAAGCACTCCTTCGCCCTGT     358
D                   AACGAGGTCACGGCGCTGGTCCCCATGCTGCACGAGGCCAAGCACTCCTTCGCCCTGT     358
                    **********************************************************
```

Figure S8. An illustration showing how the TECPR1 gene (358 bp) aligns in healthy calves (H) and those with diarrhea (D).

```
XM_010993804.3      GAGCATGCTGAGGAGTTCCACAAGCTGGGCTGCGAGGTGCTGGGGGTCTCCGTCGACTCT    60
H                   GAGCATGCTGAGGAGTTCCACAAGCTGGGCTGCGAGGTGCTGGGGGTCTCCGTCGACTCT    60
D                   GAGCATGCTGAGGAGTTCCACAAGCTGGGCTGCGAGGTGCTGGGTGTCTCCGTCGACTCT    60
                    ******************************************** ***************

XM_010993804.3      CAGTTCACCCACCTGGCTTGGATCAACACCCCTCGGAAGGAGGGAGGCTTGGGCCCCCTG    120
H                   CAGTTCACCCACCTGGCTTGGATCAACACCCCTCGGAAGGAGGGAGGCTTGGGCCCCCTG    120
D                   CAGTTCACCCACCTGGCTTGGATCAACACCCCTCGGAAGGAGGGAGGCTTGGGCCCCCTG    120
                    ************************************************************

XM_010993804.3      AACATCCCCCTGCTGGCTGATGTAACCAGAAGCTTGTCCCGTGATTATGGTGTGCTAAAG    180
H                   AACATCCCCCTGCTGGCTGATGTAACCAGAAGCTTGTCCCGTGATTATGGTGTGCTAAAG    180
D                   AACATCCCCCTGCTGGCTGATGTAACCAGAAGCTTGTCCCGTGATTATGGTGTGCTAAAG    180
                    ************************************************************

XM_010993804.3      GAAGACGAGGGCATCGCCTACAGGGGCCTCTTTATCATCGATGGCAAGGGTATCCTTCGC    240
H                   GAAGACGAGGGCATCGCCTACAGGGGCCTCTTTATCATCGATGGCAAGGGTATCCTTCGC    240
D                   GAAGACGAGGGCATCGCATACAGGGGCCTCTTTATCATCGATGGCAAGGGTATCCTTCGC    240
                    ***************** ******************************************

XM_010993804.3      CAGATCACTATCAATGATTTGCCCGTGGGACGCTCCGTGGACGAGGCTCTGCGGCTGGTC    300
H                   CAGATCACTATCAATGATTTGCCCGTGGGACGCTCCGTGGACGAGGCTCTGCGGCTGGTC    300
D                   CAGATCACTATCAATGATTTGCCCGTGGGACGCTCCGTGGACGAGGCTCTGCGGCTGGTC    300
                    ************************************************************

XM_010993804.3      CAGGCCTTCCAGTACACAGATGAGCACGGGGAAGTCTGTCCTGCTGGCTGGAAGCCAGGC    360
H                   CAGGCCTTCCAGTACACAGATGAGCACGGGGAAGTCTGTCCTGCTGGCTGGAAGCCAGGC    360
D                   CAGGCCTTCCAGTACACAGATGAGCACGGGGAAGTCTGTCCTGCTGGCTGGAAGCCAGGC    360
                    ************************************************************

XM_010993804.3      AGTGACACAATCAAGCCCAATGTGGACGACAGCAAGGAATATTTCTCCAAACACAAC       417
H                   AGTGACACAATCAAGCCCAATGTGGACGACAGCAAGGAATATTTCTCCAAACACAAC       417
D                   AGTGACACAATCAAGCCCAATGTGGACGACAGCAAGGAATATTTCTCCAAACACAAC       417
                    *********************************************************
```

Figure S9. An illustration showing how the PRDX2 gene (417 bp) aligns in healthy calves (H) and those with diarrhea (D).

```
XM_064487775.1      TGCTGGCGCTGCTCTGTGCCTACCTGCTCATGGCGACACACGCCTCGGAAGCCTGGACCA   60
H                   TGCTGGCGCTGCTCTGTGCCTACCTGCTCACGGCGACACACGCCTCGGAAGCCTGGACCA   60
D                   TGCTGGCGCTGCTCTGTGCCTACCTGCTCATGGCGACACACGCCTCGGAAGCCTGGACCA   60
                    ****************************** *****************************

XM_064487775.1      ACCCAGACCCGCAGGACCCCGGCTTCGGCATGGAGGAGCAGATCCGCGACATGCACGCCA   120
H                   ACCCAGACCCGCAGGACCCCGGCTTCGGCATGGAGGAGCAGATCCGCGACATGCACGCCA   120
D                   ACCCAGACCCGCAGGACCCCGGCTTCGGCATGGAGGAGCAGATCCGCGACATGCACGCCA   120
                    ************************************************************

XM_064487775.1      AAGTGACGGAGATCTGGCAGGAGATGAAGCAGCGGCGGGCGGCGAGTGGCCAAGATGCTG   180
H                   AAGTGACGGAGATCTGGCAGGAGATGAAGCAGCGGCGGGCGGCGAGTGGCCAAGATGCTG   180
D                   AAGTGACGGAGATCTGGCAGGAGATGAAGCAGCGGCGGGCGGCGAGTGGCCAAGATGCTG   180
                    ************************************************************

XM_064487775.1      CGCTGCACGCCGCCTGCCGCGTGCTGCCGTCGGCCGCGCTGGCCGCGGCGCAGCCCCGGG   240
H                   CGCTGCACGCCGCCTGCCGCGTGCTGCCGTCGGCCGCGCTGGCCGCGGCGCAGCCCCGGG   240
D                   CGCTGCACGCCGCCTGCCGCGTGCTGCCGTCGGCCGCGCTGGCCGCGGCGCAGCCCCGGG   240
                    ************************************************************

XM_064487775.1      TGAGGGGCCTCGTGCTCTTCCGGCAGCTCCGGCCCGGCGCCCTGCTCGAGGCCTTCTTCC   300
H                   TGAGGGGCCTCGTGCTCTTCCGGCAGCTCCGGCCCGGCGCCCTGCTCGAGGCCTTCTTCC   300
D                   TGAGGGGCCTCGTGCTCTTCCGGCAGCTCCGGCCCGGCGCCCTGCTCGAGGCCTTCTTCC   300
                    ************************************************************

XM_064487775.1      ACCTGGAGGGCTTCCCGACCGAGCCCAACGTCACCAGCC      339
H                   ACCTGGAGGGCTTCCCGACCGAGCCCAACGTCACCAGCC      339
D                   ACCTGGAGGGCTTCCCGACCGAGCCCAACGTCACCAGCC      339
                    ***************************************
```

Figure S10. An illustration showing how the SOD3 gene (339 bp) aligns in healthy calves (H) and those with diarrhea (D).

```
XM_011000575.3      CATCTAAAGGATCCAGATATGGTCTGGGACTTCTGGAGCCTGCGCCCTGAGTCTCTGCAT    60
H                   CATCTAAAGGATCCAGATATGGTCTGGGACTTCTGGAGCCTGCGCCCTGAGTCTCTGCAT    60
D                   CATCTAAAGGATCCAGATATGGTCTGGGACTTCTGGAGCCTGCGCCCTGAGTCTCTGCAT    60
                    ************************************************************

XM_011000575.3      CAGGTTTCCTTCCTGTTCAGTGACCGAGGGATTCCAGACGGTCACAGACACATGAACGGA    120
H                   CAGGTTTCCTTCCTGTTCAGTGACCGAGGGATTCCAGACGGTCACAGACACATGAACGGA    120
D                   CAGGTTTCCTTCCTGTTCAGTGACCGAGGGATTCCAGACGGTCACAGACACATGAACGGA    120
                    ************************************************************

XM_011000575.3      TACGGATCGCATACTTTCAAGCTGGTTAATGCGAATGGAGAGGCAGTTTATTGCAAATTT    180
H                   TACGGATCGCATACTTTCAAGCTGGTTAATGCGAATGGAGAGGCAGTTTATTGCAAATTT    180
D                   TACGGATCGCATACTTTCAAGCTGGTTAATGCGAATGGAGAGGCAGTTTATTGCAAATTT    180
                    ************************************************************

XM_011000575.3      CATTACAAGACTGACCAGGGCATCAAAAACCTTCCTGTTGAAGAAGCAGCAAGACTTGCC    240
H                   CATTACAAGACTAACCAGGGCATCAAAAACCTTCCTGTTGAAGAAGCAGCAAGACTTGCC    240
D                   CATTACAAGACTGACCAGGGCATCAAAAACCTTCCTGTTGAAGAAGCAGCAAGACTTGCC    240
                    ************ ***********************************************

XM_011000575.3      CAGGAAGATCCCGACTACGGCATCCGGGATCTTTTTAATGCCATTGCCACAGGCAACTAT    300
H                   CAGGAAGATCCCGACTACGGCATCCGGGATCTTTTTAATGCCATTGCCACAGGCAACTAT    300
D                   CAGGAAGATCCCGACTACGGCATCCGGGATCTTTTTAATGCCATTGCCACAGGCAACTAT    300
                    ************************************************************

XM_011000575.3      CCCTCCTGGACCTTTTACATCCAGGTCATGACATTTAATGAAGCAGAAAACTTTCCATTT    360
H                   CCCTCCTGGACCTTTTACATCCAGGTCATGACATTTAATGAAGCAGAAAACTTTCCATTT    360
D                   CCCTCCTGGACCTTTTACATCCAGGTCATGACATTTAATGAAGCAGAAAACTTTCCATTT    360
                    ************************************************************

XM_011000575.3      AATCCATTTGATCTTACCAAGGTTTGGCCTCACGGGGACTATCCTCTTATCCCAGTTGGT    420
H                   AATCCATTTGATCTTACCAAGGTTTGGCCTCACGGGGACTATCCTCTTATCCCAGTTGGT    420
D                   AATCCATTTGATCTTACCAAGGTTTGGCCTCACGGGGACTATCCTCTTATCCCAGTTGGT    420
                    ************************************************************

XM_011000575.3      AAACTGGTCTTAAACCGGAATCCAGTTAATTAC   453
H                   AAACTGGTCTTAAACCGGAATCCAGTTAATTAC   453
D                   AAACTGGTCTTAAACCGGAATCCAGTTAATTAC   453
                    *********************************
```

Figure S11. An illustration showing how the CAT gene (453 bp) aligns in healthy calves (H) and those with diarrhea (D).

```
XM_031451712.2      CTAGATGAAGAGACAGGTGAATTCCTCCCAGTTCAGCCAGCCCAACACATCCCATCAGAA   60
H                   CTAGATGAAGAGACAGGTGAATTCCTCCCAGTTCAGCCAGCCCAACACATCCCATCAGAA   60
D                   CTAGATGAAGAGACAGGTGAATTCCTCCCAGTTCAGCCAGCCCAACACATCCCATCAGAA   60
                    ************************************************************

XM_031451712.2      ACCAGTGGATCTACCAACTACTCCCAGGTTTCTTCGGCTACGTTTCAATCACTTGTTCCT   120
H                   ACCAGTGGATCTACCAACTACTCCCAGGTTTCTTCGGCTACGTTTCAATCACTTGTTCCT   120
D                   ACCAGTGGATCTACCAACTACTCCCAGGTTTCTTCGGCTACGTTTCAATCACTTGTTCCT   120
                    ************************************************************

XM_031451712.2      GATATTCCTAGCCACATCCAGAGCCCCGTCTTCACTGCTCCTAATCAGGCTGAGTCACCT   180
H                   GATATTCCTAGCCACATCCAGAGCCCCGTCTTCACTGCTCCTAATCAGGCTCAGTCACCT   180
D                   GATATTCCCAGCCACATCCAGAGCCCCGTCTTCACTGCTCCTAATCAGGCTGAGTCACCT   180
                    ******** ****************************************** ********

XM_031451712.2      GAAACTCTTGTCCTTCAGTTAGCCACTACTGATTTAGACGCTATGCAGCGAGATTTTGAG   240
H                   GAAACTCTTGTCCTTCAGTTAGCCACTACTGATTTAGACGCTATGCAGCGAGATTTTGAG   240
D                   GAAACTCTTGTCCTTCAGTTAGCCACTACTGATTTAGACGCTATGCAGCGAGATTTTGAG   240
                    ************************************************************

XM_031451712.2      CAAGTTTGGGAGGAGCTATTATCCATTCCAGAACTACAGTGTCTTAATATTCAAAATGAC   300
H                   CAAGTTTGGGAGGAGCTATTATCCATTCCAGAACTACAGTGTCTTAATATTCAAAATGAC   300
D                   CAAGTTTGGGAGGAGCTATTATCCATTCCAGAACTACAGTGTCTTAATATTCAAAATGAC   300
                    ************************************************************

XM_031451712.2      AACCTGGCTGAGACTAGCACAGTTCCAAGTCCAGAAACCAAACTGACAGAAATGGACAGT   360
H                   AACCTGGCTGAGACTAGCACAGTTCCAAGTCCAGAAACCAAACTGACAGAAATGGACAGT   360
D                   AACCTGGCTGAGACTAGCACAGTTCCAAGTCCAGAAACCAAACTGACAGAAATGGACAGT   360
                    ************************************************************

XM_031451712.2      TACTTCTATTCATCAATCCCCTCACTGGAAAAAGAAGTAGGTAACTGTAGTCCACAT      417
H                   TACTTCTATTCATCAATCCCCTCACTGGAAAAAGAAGTAGGTAACTGTAGTCCACAT      417
D                   TATTTCTATTCATCAATCCCCTCACTGGAAAAAGAAGTAGGTAACTGTAGTCCACAT      417
                    ** ******************************************************
```

Figure S12. An illustration showing how the Nrf2 gene (417 bp) aligns in healthy calves (H) and those with diarrhea (D).
